# Surface Electromyography in Physiotherapist Educational Program in France: Enhancing Learning sEMG in Stretching Practice

**DOI:** 10.3389/fneur.2020.584304

**Published:** 2020-12-03

**Authors:** Pierre Portero, Anton A. Dogadov, Christine Servière, Franck Quaine

**Affiliations:** ^1^Université Paris–Est Créteil, Faculté de santé, équipe Bioingénierie, Tissus et Neuroplasticité (BIOTN), Créteil, France; ^2^Université Grenoble Alpes, Centre National de la Recherche Scientifique, Inria, Grenoble INP, GIPSA-lab, Grenoble, France; ^3^Université Paris-Saclay, Centre National de la Recherche Scientifique, Paris-Saclay Institute of Neuroscience (NeuroPSI), Gif-sur-Yvette, France

**Keywords:** surface electromyography (EMG), stretching, biofeedback, *triceps surae*, physiotherapy

## Abstract

Surface electromyography (sEMG) is a non-invasive method, which may be used in France by health practitioners without medical degree, such as physiotherapists, who are taught in Institutes of physiotherapy. However, very few hours are devoted to sEMG teaching in physiotherapist educational programs, especially in a form of practical work. In order to motivate using sEMG in physiotherapy to the students, we propose an example of sEMG practical work, applied to muscle stretching. Passive stretching exercises are often used by physiotherapists to maintain or improve range of motion. During a passive stretching session, subjects are given specific instructions to relax and not to activate their muscles during the procedure. In the proposed practical work, the sEMG is used to study the plantar flexor activation level during passive stretching. Therefore, this work may provide students with deeper understanding of physiology and biomechanics, trigger an interest in sEMG as a tool, and give knowledge about good sEMG practice, according to SENIAM and other recommendations. The integration of Institutes of physiotherapy in the University system may provide an opportunity to revisit the physiotherapist educational program and to provide students with more practical courses on sEMG application.

## Introduction

The clinical use of EMG in France for neuromuscular diseases diagnosis is performed by medical doctors using needle EMG only. However, according to French Common Classification of Medical Acts [CCAM, (1)] nomenclature, “sEMG can provide useful information different from the needle EMG technique, since it is an authorized method, which may be applied according to the physiotherapist's choice without increasing overall cost of the physiotherapy act, refunded by National Insurance [(2) and following, concerning physiotherapists, giving them the liberty of technic choice]. sEMG is a non-invasive technique, which may be used by health practitioners without medical degree, such as physiotherapists.

Starting from 2015, physiotherapist educational program duration in France is 4 years in Institutes of physiotherapy, preceded by 1 year of either medical study, sport-science studies or natural/formal science bachelor programs, i.e., overall education time is 5 years after high school (3, 4). The students of these programs, who successfully passed the ranking, can enter one of 46 (as for 2017 according to French Association of masseur-physiotherapists) public or private Institutes of physiotherapy, according to their ranking (5). All the private institutes are independent structures, affiliated with a University by an agreement. Nevertheless, some public Institutes of physiotherapy are integrated with Universities (Paris-Est Créteil, Orléans, Limoges, Grenoble). Graduating from Institutes of physiotherapy provides a student with a national diploma, which is not an academic degree. After graduating, physiotherapists can continue their studies by applying for a master science program in movement science, kinesiology, or biomechanics, which may be also organized in the format of double diploma (master science – physiotherapy).

The recommended educational program for Institutes of physiotherapy contains a course about the theories, models, methods, and tools in physiotherapy with a total volume of 40 h of lectures and 40 h of tutorials. This course is conducted during four first semesters. EMG is cited among the other methods, like motion capture, force plate and gauge, inertial sensors as the methods recommended to be presented in this course.

To our knowledge based on the inquiry among French Institutes of physiotherapy, the time devoted to teaching musculoskeletal EMG is little (among seven institutes that we contacted only two give a class on EMG without practical work, and the others do not give a specific class on it).

One of the main parts of physiotherapist practice is to recover or maintain the maximal range of motion which may be performed by passive mobilization or stretching, performed by a physiotherapist or a patient under the supervision of the former. The ability to move efficiently through a large range of motion (ROM) is essential for the successful completion of numerous tasks in daily living, work, and sports. Stretching exercises are often advocated to maintain and improve ROM (6). Optimal passive stretching techniques require the muscles to be inactivated as much as possible. This state involves a physiological muscle resting state, which is challenging to reach for the subjects.

## Laboratory Studies Showing the Muscular Activation During Stretching

There is a number of studies, aimed to quantify the muscle activation level during stretching, when the subjects were asked to maintain a relaxed state (7–9). For that purpose, sEMG is often measured from the plantar flexor muscles and the root mean square (RMS) of the signal is usually normalized to signal RMS during isometric maximal voluntary contractions (MVC) of these muscles. These studies show that during an initial stretch produced by individuals who have not stretched for long time, sEMG responses above 2% of MVC RMS appear for joint angle starting from 80% of the maximal range of motion. These sEMG responses increase as the joint angle approaches the maximum tolerated stretch amplitude. The sEMG can vary between 2 and 15% of MVC RMS and is more frequently observed in the older adult. With repeated stretching, this activity usually decreases considerably below 2% of MVC RMS.

The recent study (9) has quantified the muscle mechanical changes, when the muscles were voluntarily activated at 1, 2, and 5% of MVC RMS, with reference to “relaxed” conditions, where the subject was asked to produce no voluntary activation. During the experiment, the ankle was dorsiflexed using isokinetic dynamometer, and the participants were asked to produce low muscle activation using a visual feedback of sEMG amplitude at 1, 2, and 5% of maximal sEMG or to stay fully passively relaxed as possible during stretching. The results show a significant increase in joint torque (+ 33%) and muscle shear modulus (+ 55, + 38, and + 100%, for *gastrocnemius medialis, gastrocnemius lateralis*, and *soleus*, respectively), when the participants activated their muscles at 5% activation during slow dorsiflexions of the ankle. Nevertheless, even at a lower activation level of sEMG amplitude (2%) the change in joint torque was significant (+14%).

## Educational Application

**Figure 1:**
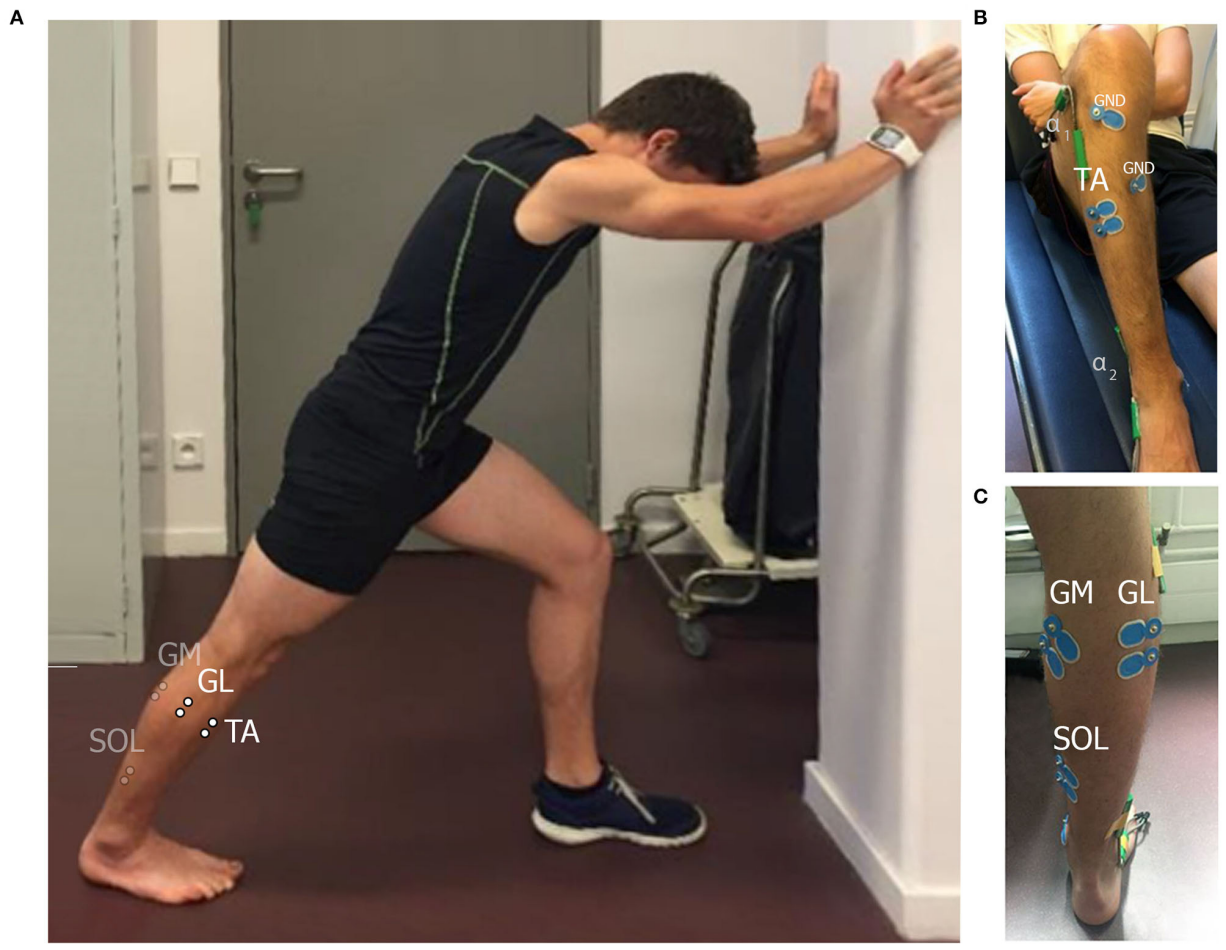
Wall stretching; **(A)** standing position. Differential electrode placements are schematically shown for gastrocnemius medialis (GM), gastrocnemius lateralis (GL), soleus (SOL), and tibialis anterior (TA) muscles. Ground electrode placements are indicated by GND. In this example, an individual ground electrode was used for each pair of differential electrodes. Goniometer placement sites are indicated by α_1_ and α_2._ **(B,C)** Show the ventral and the dorsal view of the leg with placed electrodes correspondingly.

One of the main stretching methods commonly used in clinical practice is called “wall stretching” (Figure 1). Wall stretching is performed by placing the foot at a distance from the wall, with the subject leaning forward, keeping the knee in extension, which leads to stretching the TS muscle (10).

We propose to integrate the practical work on sEMG into the physiotherapist educational program in order to demonstrate the interest on sEMG application in practice related to force application on passive structures.

**Figure 2:**
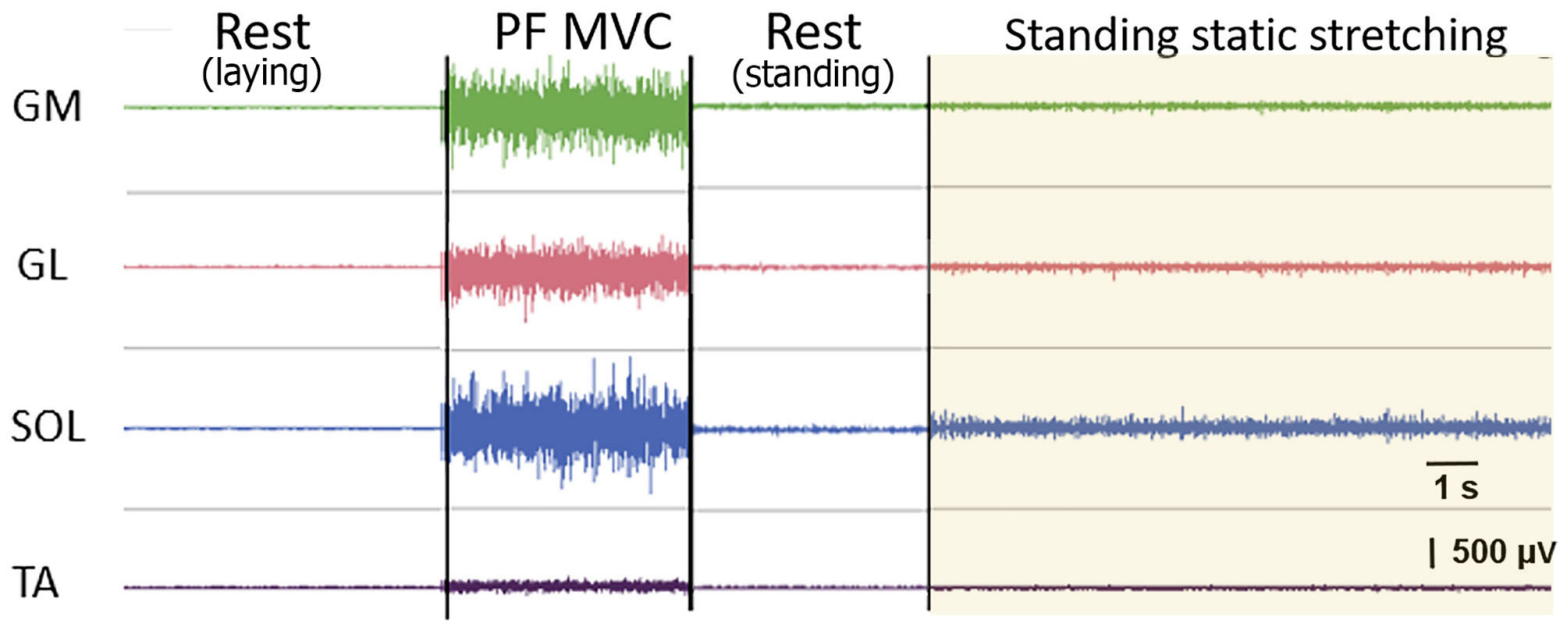
Typical raw electromyogram signals of gastrocnemius medialis (GM), gastrocnemius lateralis (GL), soleus (SOL), and tibialis anterior (TA) muscles during different stages of the proposed protocol: at rest, during a plantarflexion maximal voluntary contraction (plantar flexor MVC), and during a static stretching. A slight signal recorded from TA can be either an activity of this muscle or a crosstalk from *triceps surae* muscles.

This work may be performed in pairs: one student in a role of an experimental subject and the second one as an investigator. The investigator places the surface electrodes and the goniometers as shown in Figure 1. The electrodes are placed on *gastrocnemius medialis, gastrocnemius lateralis*, and *soleus* muscles (GM, GL, and SOL), which are plantar flexors, forming *tripces surae* muscles, and on *tibialis anterior* muscle (TA) according to SENIAM recommendations (11). The goniometers are placed above ankle and knee joints. The investigator asks a subject to lay down and records the sEMG in rest. Next, the subject is asked to stand up in front of the wall in a position, shown in Figure 1A to measure the RMS of sEMG during plantar flexor MVC. After a resting period (at least 5 min) a subject is asked to perform a passive wall stretching protocol for 30 s at 80% of maximal ROM of the ankle and full knee extension. The corresponding angles are measured with goniometers and displayed to the subject. The typical signals, recorded from the sEMG electrodes during all stages of the protocol, described above, are shown in Figure 2. An involuntary activity of the muscles, forming *tripces surae* muscles may be noticed during passive stretching phase. A slight signal recorded from TA can be either an activity of this muscle or a crosstalk from *triceps surae* muscles. After completing the protocol, the roles of investigator and experimental subject can be inverted.

The questions asked to the students are:

- Which muscles are activated during stretching?
- What was the observed activation level of stretched muscles in percentage of root-mean-square of sEMG from corresponding muscle during MVC?
- Was the stretching completely passive?

The main limitation of this work is the availability of the experimental setup. If only one set of measurement equipment is available, this session may be performed in 2 h by two pairs if the roles are switched or by five pairs without switching the roles. At the end of the session, the pairs are encouraged to share the results and discuss them before writing the group report.

## Conclusion and Perspectives

In this paper we propose an example of a practical work which is aimed to provide physiotherapists students with a deeper understanding of the neuromuscular physiology and biomechanics of stretching. This work also shows the value of sEMG as a biofeedback, which is an informative tool, not widely used in neuromuscular rehabilitation in France. Finally, this work might make the student familiar with the recommendation about the sEMG procedure, such as SENIAM recommendations (11) and CEDE project (12). These skills may be used by future physiotherapists not only in a given stretching example, but also in other applications.

The recent extension of the physiotherapist studies in France (from 4 to 5 years) might give an opportunity to develop the educational programs in scientific areas. Due to this educational reform, relevant sEMG application courses may be integrated into the French physiotherapist academic programs. Currently (13), French Health Ministry and Ministry of Higher Education and Research start an experimental educational program, including some universities, in order to approach French Institutes of physiotherapy with Universities. This integration may be also used to revisit the physiotherapist educational program and to provide students with more practical courses on sEMG application.

At the same time, further development of sEMG equipment could improve its usability, such as textile-based electrode arrays, which do not require precise electrode placement; capacitive electrodes, which do not require skin treatment; wireless sensors, which do not restrain movement. We believe that improving the usability of sEMG, together with available professional training program will lead practitioners to use of sEMG in their practice.

## Data Availability Statement

The raw data supporting the conclusions of this article will be made available by the authors, without undue reservation.

## Ethics Statement

Ethical review and approval was not required for the study on human participants in accordance with the local legislation and institutional requirements. The patients/participants provided their written informed consent to participate in this study. Written informed consent was obtained from the individual(s) for the publication of any potentially identifiable images or data included in this article.

## Author Contributions

All authors listed have made a substantial, direct and intellectual contribution to the work, and approved it for publication.

## Conflict of Interest

The authors declare that the research was conducted in the absence of any commercial or financial relationships that could be construed as a potential conflict of interest.
